# Book Review – For the common good

**DOI:** 10.1177/17407745231193140

**Published:** 2023-08-23

**Authors:** Søren Holm

Over the last two decades, Alex John London has been one of the most thoughtful and distinctive contributors to debates about biomedical research ethics. This book builds on and extends his previous work and presents an extended argument for a basic reconfiguration of research ethics.

Based on a philosophical reading of the history of research ethics, London argues that we have ended up with a problematically narrow view of what the basis for research ethics is, and what research ethics systems should try to do. He calls this the ‘IRB triangle’ where the focus is on:

‘… the discrete interactions of researchers and participants that are reflected in study protocols; informed consent forms; and that are evaluated by an Institutional Review Board (IRB)’. (p 7)

London argues that confining research ethics to the IRB triangle leads to a situation where the wider context, including important questions about justice are ignored because they fall outside of the triangle.

The constructive part of London’s extended argument is based on the identification of the knowledge generated by research as a ‘common good’ in the sense used in economic theory, that is, a good that is non-rivalrous and non-excludable. As soon as knowledge has been generated and made public, it can be used by anyone. Given that societies have public health and medical challenges that can be addressed through the generation of knowledge this then entails that we can conceptualise research as a social cooperative activity aimed at filling these important knowledge gaps. From this, we can then argue for The Egalitarian Research Imperative that London defines in the following way:

There is a strong social imperative to enable communities to create, sustain, and engage in research understood as a scheme of social cooperation that respects the status of stakeholders as free and equal and that functions to generate information and interventions needed to enable their basic social systems to equitably, effectively, and efficiently safeguard and advance the basic interests of their constituent members. (p 18)

This specific social cooperative scheme should then be seen as a constituent part of a just society.

Borrowing insights from game theory, London also argues that research and research participation should be seen as a ‘stag hunt’. This is a collaborative scheme where individual participants gain more by participating than by not participating. In the original set-up of the game, each of us can either hunt a hare, or we can come together collaboratively to hunt a stag. Because the individual share of the stag is worth more than a hare and because collaboration is necessary to hunt the stag effectively, it becomes rational for self-interested individual to join the stag hunt.

London’s critical and constructive arguments are detailed and convincing and in the later parts of the books he shows how they can be applied to and have implications for a number of the main, seemingly intractable controversies in research ethics, for example, issues concerning exploitation, acceptable research risk, and the ‘double standard’ debate among others.

There are, however, areas in the basic constructive argument where London could have dug a little deeper. These can perhaps best be brought out by considering (1) the identification of research knowledge as a common good, (2) the nature of the research stag hunt, and (3) the index of the book.

It is true that published research knowledge is non-rivalrous and non-excludable and, therefore, definitionally a common good, but in those sectors of research where the knowledge forms the basis for protected intellectual property many of the straightforward potential uses of the knowledge are excludable. A patent discloses the knowledge, but it also creates time limited rights to exclude others from using the knowledge for the purposes described in the patent. It is worth considering whether that changes the strength or the scope of the ‘strong social imperative’ that London argues for.

A similar issue arises in relation to the stag hunt analysis. In the initial formulation of the ‘stag hunt’ in game theory, it is a two player game. When modelling repeated stag hunts in a randomly mixing population, it is found that a variety of strategies will develop and that both collaboration and non-collaboration can become the dominant strategy depending on the exact parameters of the model. But, the biomedical research stag hunt is not the stag hunt of game theory. The stag can only successfully be hunted if a large group of people collaborate; the research stag hunt usually has an undisputed leader; the payout matrix for collaborators is not uniform, some get much more of the stag than others and some participants only receive their share of the stag a long time after the successful hunt if ever. There will also be research stag hunts where the stag is successfully hunted down, but where nothing of value is distributed to most of the participants in the hunt. It is true that a successful research stag hunt, that is, a successful research project always generates knowledge since that is definitional for successful research, but those who participate solely as research participants may well have expectations that this knowledge will be utilised to the benefit of them or people like them and they may decide to participate based on those expectations and not purely based on a willingness to participate in a knowledge-generating scheme of social cooperation. This means that not all participants in the stag hunt are actually hunting the same stag or have the same expectations about how the stag should be distributed after the hunt.

I won’t put too much weight on the final critical point, but perhaps the index of the book is also telling. There are no entries for terms like industry, patent, profit, or power. The book has many insightful analyses of asymmetries between researchers, research participants, and communities, but could perhaps have dug a little deeper into the causes that create and sustain these asymmetries.

This is, despite the critical comments above undoubtedly one of the most important books about biomedical research ethics to appear in the last 20 years. Anyone with an interest in research ethics or a role in planning, funding, or regulating research ought to read it, and engage with both the critical and the constructive argument.


Søren Holm The University of Manchester, Manchester, UK University of Oslo, Oslo, Norway


